# BCMab1-Ra, a novel immunotoxin that BCMab1 antibody coupled to Ricin A chain, can eliminate bladder tumor

**DOI:** 10.18632/oncotarget.13504

**Published:** 2016-11-22

**Authors:** Chong Li, Ruping Yan, Zhao Yang, Haifeng Wang, Ruiyun Zhang, Haige Chen, Jiansong Wang

**Affiliations:** ^1^ Core Facility for Protein Research, Institute of Biophysics, Chinese Academy of Sciences, Beijing 100101, China; ^2^ Department of Urology, The Second Affliated Hospital of Kunming Medical University, Kunming 650101, China; ^3^ CAS Key Laboratory of Pathogenic Microbiology and Immunology, Institute of Microbiology, Chinese Academy of Sciences, Beijing 100101, China; ^4^ Department of Urology, Renji Hospital Affiliated to Shanghai Jiaotong University, School of Medicine, Shanghai 200127, China; ^5^ Beijing Jianlan Institute of Medicine, Beijing 100190, China

**Keywords:** BCMab1, BCMab1-Ra, bladder cancer, aberrantly glycosylated integrin a3b1

## Abstract

Bladder cancer is one of the most common malignancies. However, there is no ideal therapy to cure bladder cancer so far, especially invasive carcinoma. Here, we developed a new antibody-based drug BCMab1-Ra, which was generated by conjugation of BCMab1 (a new monoclonal antibody that specifically recognized the aberrantly glycosylated Integrin a3b1 in bladder cancer) with the ricin A chain (Ra). A patient with multiple bladder cancer received intravescical administration of BCMab1-Ra treatment as a volunteer. After 30 weeks of treatment, no tumor was observed by cystoscope examination. We did not observe any local or systemic side effects. Human anti-mouse antibody (HAMA) was not detectable in the circulation. Results follow-up showed no tumor had been found in every half year review in 3 years.

In July, 2011, a 57-year-old man presented to us with gross hematuria. Cystoscope examination showed multiple tumors on the left- bottom wall of bladder. There were five tumors of different sizes without extensively invading from the bladder neck, to the posterior wall, the right lateral wall, and the apex of the bladder. The largest is more than 5 cm, others are from 1 to 2 cm. The largest tumor was diagnosed to be Grade III transitional cell carcinoma (TCC) by biopsy and from the above findings, it was diagnosed as invasive bladder tumor with a clinical stage of T3, N0, M0.

The patient refused radical surgery and chemotherapy. He agreed to receive intravescical infusion of BCMab1-Ra treatment as a volunteer. The preliminary clinical study was started under the agreements of the patient and the institutional review board of the hospital, and informed consent was obtained and documented in writing from the patient. BCMab1 is a new specific monoclonal antibody which specifically recognized the aberrantly glycosylated Integrin a3b1 in bladder cancer [1, 2]. BCMab1-Ra was generated by conjugation of BCMab1 with the ricin A chain (Ra) as an immunotoxin. 2 ml BCMab1-Ra (40 mg/ml) diluted in 40 ml normal saline (NS), and then was infused into the bladder through a urethral catheter. The patient kept the solution in the bladder for at least 1 hour and turned 90 degrees every 15 min. He received BCMab1-Ra weekly for 10 weeks, then biweekly for 20 weeks. We examined the patient's blood chemistry and urine tests before treatment and every week during the treatment to evaluate systemic adverse effects.

After 4 weeks of treatment, no hematuria appeared. 26 weeks later, it's surprising that the tumors were disappeared by cystoscope examination. In the course of treatment, we did not observe any local or systemic side effects associated with administration of BCMab1-Ra (Figure 1). Human anti-mouse antibody (HAMA) was not detectable in the circulation. Results Follow-up showed no tumor had been found in every half year review.

**Figure 1:**
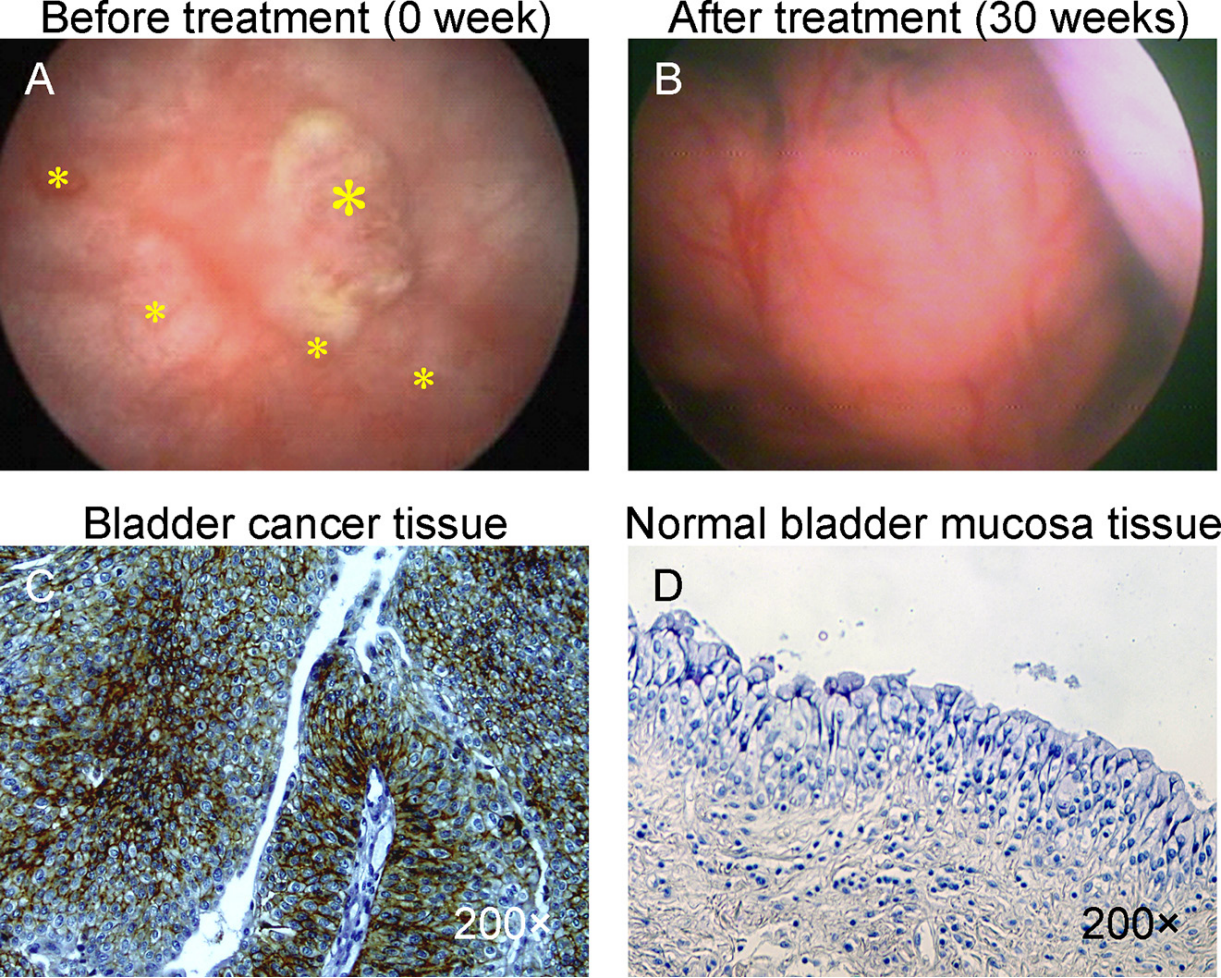
Photograph of bladder cancer with intravescical administration of BCMab1-Ra (**A**) Cystoscope photo before treatment with BCMab-1Ra. (**B**) Cystoscope photo after treatment with BCMab1-Ra. BCMab1 recognized its antigen on the bladder cancer tissue (**C**), bot not on the normal bladder mucosa tissue (**D**).

Local delivery of BCMab1-Ra can avoid many problems associated with systemic therapy, such as (i) absorption by the mononuclear phagocyte system (mainly the liver and spleen) during antibody catabolism; (ii) only a small amount of antibodies reaching the inside tumor and (iii) localization of antibodies in normal organs and formation of human anti-mouse antibodies (HAMA). BCMab1-Ra may serve as a novel specific antibody-based drug for targeted therapy of bladder cancer.
